# Editorial: Natural products as sources of innovative approaches in psychiatry

**DOI:** 10.3389/fpsyt.2022.929139

**Published:** 2022-07-26

**Authors:** Elaine Elisabetsky, Chun-Tao Che

**Affiliations:** ^1^Department of Biochemistry, Federal University of Rio Grande do Sul, Porto Alegre, Brazil; ^2^College of Pharmacy, University of Illinois at Chicago, Chicago, IL, United States

**Keywords:** natural products, mental disorders, drug development, innovative antidepressants, medicinal plants revisited

Between 1981 and 2010, more than thirty percent of the new medicines approved by the US FDA were based on natural products and/or their derivatives. Advantages of natural products in terms of drugability and bioactivity have been brilliantly reviewed and summarized by Harvey et al. (1) and Skirycz et al. (2), in part explaining why the vast majority of drugs have a close match with natural products (1). Today we also witness global interest in the use of plant-based therapies and botanical healthcare products. Such a trend has stimulated greater scientific awareness in exploring the pharmacologically active constituents of medicinal plants. This Special Issue is intended to be a compilation of scientific reports to demonstrate the potential of natural products for the management of psychiatric disorders. It is our hope that this Special Issue would serve those researchers who are interested in potentially useful molecules from natural sources for psychiatric applications.

The anti-depressant-like effects of natural products and herbal preparations were demonstrated by a series of studies.

Zhou et al. reported a Chinese herbal drug (Jie-Yu Pill) to be able to ameliorate mood disorder-like behavior and cognitive impairment using a mouse model induced by a combination of estrogen deprivation and chronic stress, implying the potential of Jie-Yu Pill in managing menopause-associated mood disorders.

Studies on depression-like behaviors and cognitive dysfunction was extended to microgravity and social isolation scenarios. Wang et al. reported that dammarane sapogenins (obtained from ginseng) could reverse the depressive-like behaviors and improve cognitive impairment in rats, and concluded that the protective effects might be driven in part by the modulation of cholinergic system in the hippocampus.

In a study by Ayuob et al., *Ocimum basilicum* essential oil was given to mice under chronic unpredictable mild stress (CUMS) in order to assess the effectiveness of the preparation on the main olfactory bulb (MOB). Their results revealed improvements in both biochemical and histopathological changes in the MOB. They suggested the effect might have been attained through an up-regulation of gene expression of GFAP and Ki67 and down-regulation of Caspase-3 in the MOB.

Jiang et al. reported the protective effect of a Chinese herbal drug (Shen Yuan, composed of *Panax ginseng* and *Polygala tenuifolia*) in the rat chronic unpredictable mild stress model. Shen Yuan reversed the depressive-like behaviors as well as biochemical depressive states correlates, including increased serum corticosterone and proinflammatory cytokines (IL-6, IL-1β, and TNF-α) levels, oxidative stress markers (SOD, CAT, and MDA), and diminished levels of hippocampal neurotransmitters (5-HT, DA, and NE) in rats exposed to CUMS. Furthermore, rats treated with Shen Yuan showed reduced hippocampus BDNF, p-TrkB, p-Akt, and p-mTOR proteins expression raised by CUMS exposure. The findings pointed to a preventive effect of Shen Yuan against hypothalamus-pituitary-adrenal axis dysfunction, decreased in neurotransmitters levels, reduced oxidative stress, neuroinflammation suppressio, and activation of the PI3K/Akt/mTOR-mediated BDNF/TrkB pathway. All of these effects were thought to be relevant to counteract depressive states.

Curcumin has been reported to exert beneficial effects on managing major depressive disorder. Highlights of the compound's clinical and non-clinical effects are presented in a review by Ramaholimihaso et al.

A significant amount of attention has recently been given to the potential therapeutic value of endogenous modulators.

Still on depression, Almeida et al. reported the antidepressant-like effects of chronic guanosine in the olfactory bulbectomy (OBX) mouse model. Guanosine reversed the OBX-induced recognition memory impairment and hyperlocomotion, increase in hippocampal BDNF and redox imbalance. Guanosine also mitigated the OBX-induced hippocampal neuroinflammation and increased metabolism.

Tonon et al. reviewed the evidences of melatonin for managing depressive states, noting that clinical evidence was inconsistent in regard to the benefits of melatonin or melatonin agonists in fighting depression, in contrast with the antidepressant-like effects observed in animal models. The authors argue that the understanding of melatonin in therapeutics must include melatonin specificities as an integrating molecule, associated with entrainment, metabolism, immunity, neurotransmission, and cell homeostasis.

Bitencourt et al. reviewed the path that led to the development of cannabidiol as an antiepileptic drug, highlighting early contributions by Brazilian scientists. Authors elaborated on the idea that CDB development model can be used to develop phytocannabinoids for other psychiatric conditions, including depression, anxiety, post-traumatic stress disorder (PTSD), addiction, neurodegenerative disorders and autism spectrum disorder (ASD).

Cognitive-enhancing effects of the Chinese medicinal plant, *Dendrobium nobile*, in sleep deprivation-induced amnesia in mice was reported by Jiang et al.. The results suggest amnesia was improved by the treatment in the novel object recognition and object location recognition tests. They also reported elevated levels of norepinephrine, dismutase and catalase activities, as well as a decrease in 5-HT and malondialdehyde, in the brain tissue obtained from the *Dendrobium* treated group. The authors conclude that *D. nobile* extract has beneficial effects in the prevention and improvement of cognitive impairment induced by sleep deprivation, effects possibly mediated through the regulation of neurotransmitters and alleviation of oxidative stress.

Bian et al. reviewed clinical and pre-clinical results of saffron (dried stigma of *Crocus sativus*) and its constituents (crocin, crocetin and safranal) on brain disorders. The review covers a range of pathologies such as depression, anxiety, Alzheimer's and Parkinson's diseases, post-traumatic stress disorder, schizophrenia, epilepsy, and stroke. Preclinical studies showed that saffron exerts its neuroprotective effects mostly via antioxidative stress, anti-neuroinflammation, and anti-apoptosis pathways. Clinical results supported that saffron could alleviate depressive and anxiety-like symptoms as well as improve cognition impairment. The authors suggested that these findings had provided a clear perspective that could aid the development of neuroprotective agents from saffron and its bioactive ingredients.

Zhao et al. suggest that bulleyaconitine A (BAA), a C19-diterpenoid alkaloid used in China for decades as nonnarcotic analgesic to treat chronic pain, is a candidate for treatment of opioids addiction. They showed that BAA attenuates morphine-induced withdrawal symptoms in mice, conditioned place preference and locomotor sensitization by stimulation of microglial (but not astrocytes or neurons) dynorphin A expression in nucleus accumbens and hippocampus.

Marx et al. reported a secondary analysis of a randomized placebo-controlled trial that investigated a 24-weeks intervention with mangosteen (*Garcinia mangostana* Linn.) pericarp extract supplementation in people diagnosed with schizophrenia. The secondary analysis investigated if the intervention was effective to improve cognition in this population and revealed that mangosteen pericarp extract did not affect cognitive outcomes in people with schizophrenia.

In conclusion, the above studies illustrates the potential of natural products against various psychiatric conditions. As usual in drug development, to better characterize and understand their medicinal properties, additional preclinical and clinical investigations are warranted.

## Author contributions

EE and C-TC contributed equally to the concept and execution of this editorial. All authors contributed to the article and approved the submitted version.

## Conflict of interest

The authors declare that the research was conducted in the absence of any commercial or financial relationships that could be construed as a potential conflict of interest.

## Publisher's note

All claims expressed in this article are solely those of the authors and do not necessarily represent those of their affiliated organizations, or those of the publisher, the editors and the reviewers. Any product that may be evaluated in this article, or claim that may be made by its manufacturer, is not guaranteed or endorsed by the publisher.
